# A discrete subpopulation of PFC-LHb neurons govern cocaine place preference

**DOI:** 10.1038/s41398-024-02988-8

**Published:** 2024-07-02

**Authors:** Nur Abdel-Hay, Marina Kabirova, Rami Yaka

**Affiliations:** Institute for Drug Research (IDR), School of Pharmacy, Faculty of Medicine, The Hebrew University of Jerusalem, Jerusalem, Israel

**Keywords:** Molecular neuroscience, Psychiatric disorders

## Abstract

Addiction is a complex behavioral disorder characterized by compulsive drug-seeking and drug use despite harmful consequences. The prefrontal cortex (PFC) plays a crucial role in cocaine addiction, involving decision-making, impulse control, memory, and emotional regulation. The PFC interacts with the brain’s reward system, including the ventral tegmental area (VTA) and nucleus accumbens (NAc). The PFC also projects to the lateral habenula (LHb), a brain region critical for encoding negative reward and regulating the reward system. In the current study, we examined the role of PFC-LHb projections in regulating cocaine reward-related behaviors. We found that optogenetic stimulation of the PFC-LHb circuit during cocaine conditioning abolished cocaine preference without causing aversion. In addition, increased c-fos expression in LHb neurons was observed in animals that received optic stimulation during cocaine conditioning, supporting the circuit’s involvement in cocaine preference regulation. Molecular analysis in animals that received optic stimulation revealed that cocaine-induced alterations in the expression of GluA1 subunit of AMPA receptor was normalized to saline levels in a region-specific manner. Moreover, GluA1 serine phosphorylation on S845 and S831 were differentially altered in LHb and VTA but not in the PFC. Together these findings highlight the critical role of the PFC-LHb circuit in controlling cocaine reward-related behaviors and shed light on the underlying mechanisms. Understanding this circuit’s function may provide valuable insights into addiction and contribute to developing targeted treatments for substance use disorders.

## Introduction

Addiction is a neurobiological illness characterized by repetitive substance abuse that disrupts the normal circuitry governing rewarding and adaptive behaviors, leading to drug-induced neuroplastic changes [1]. The principal components of the reward system, including the ventral tegmental area (VTA), nucleus accumbens (NAc), and prefrontal cortex (PFC), serve as critical substrates for the neuronal adaptations underlying addiction. Interactions between addictive drugs and synaptic plasticity in various brain regions contribute to specific aspects of addiction, such as craving, withdrawal, and relapse [2, 3]. Among the drugs that activate the reward system is the psychostimulant cocaine. The prefrontal cortex (PFC) is a crucial glutamatergic brain region responsible for higher-order cognitive functions, including executive functions, decision-making, impulse control, and judgment [4]. Studies have demonstrated that dysfunction in the PFC can contribute to addictive behaviors and drug abuse. Notably, previous studies have revealed that PFC neurons project directly into the lateral habenula (LHb) [5, 6]. The LHb is a glutamatergic region highly involved in reward processing. It has been suggested that the LHb is a major source of negative reward-related signals which contributes to the modulation of dopamine neurons activity [7] and its activation strongly inhibits dopamine neurons in the VTA [8]. Studies have shown that selective activation of PFC-LHb projecting neurons produces distinct, rapid, and reversible effects on the selection of the active behavioral state [6, 9]. Furthermore, this activation has been found to reduce sociability without inducing aversion or locomotor hyperactivity [6]. In addition, deep brain stimulation (DBS) in the LHb effectively decreased cocaine-seeking behavior, both during self-administration and extinction training [10]. However, the source of cognitive and affective input to the LHb, which is essential for its modulation of midbrain monoaminergic systems, remains unclear. The PFC is a plausible candidate for providing this input, given its control on higher-order cognitive functions.

Drugs of abuse can have distinct effects on various subpopulations of neurons within a particular brain region [11, 12]. The primary changes that occur with exposure to drugs of abuse involve the modification of fundamental types of synaptic plasticity, specifically long-term potentiation (LTP) and long-term depression (LTD) [13–15]. One of the key protein families believed to be involved in the processes of memory acquisition and maintenance is the ionotropic glutamate receptors (iGluRs) [16, 17]. Two primary types of iGluRs play pivotal roles in these processes: NMDA (N-methyl-D-aspartate), and AMPAR (alpha-amino-3-hydroxy-5-methyl-4-isoxazolepropionic acid) receptors. NMDA receptors are responsible for initiating synaptic changes [18], while AMPA receptors are involved in the maintenance of long-term changes [19]. The GluA1 subunit of the AMPA receptor plays a pivotal role in mediating excitatory neurotransmission in the brain [20, 21]. Phosphorylation of specific serine residues on the intracellular tail of GluA1 by different kinases such as PKA, that directly phosphorylates GluA1 subunit of AMPA receptors at S845 [16] and activates ERK signaling, resulting in increased pERK levels [22] or S831 governed by the CaMKII (Calcium/Calmodulin-Dependent Protein Kinase II) [13, 23], is known to be of critical importance in regulating the trafficking and function of GluA1. These phosphorylation events can influence the receptor’s localization to synapses and its overall synaptic efficacy [13, 20].

Considering the pivotal role of the LHb in higher-level cognitive control of reward-related behaviors, and the responsibility of the PFC for higher-order cognitive functions, our present study sought to investigate the impact of selectively activating PFC projections to the LHb on a reward-related behavior, specifically cocaine preference, employing a place preference paradigm and examined the effect of PFC-LHb circuit activation on GluA1 expression and phosphorylation state in the PFC, LHb and VTA.

## Materials and methods

### Animals

C57BL/6JOlaHsd male mice (16–18 g). Mice were group-housed in the animal facility at an ambient temperature of 22 °C and a 12-h light/dark cycle with the lights on at 7:00 a.m. Food and water were provided ad libitum. All procedures approved by the Institutional Animal Care Committee (IACUC) of the Hebrew University (Jerusalem, Israel). Ethics approval and consent to participate for human studies is not applicable in the current study.

### Stereotactic surgery

Mice were anesthetized with ketamine plus dormitor (0.15:0.85). Microinjection needle (33 gauge) connected to a 10 µl syringe (Hamilton) were then inserted unilaterally directly to the PFC (AP: +2.1, ML: 0.4, DV: −1.2).0.5 µl of a purified AAV (2 × 10^12^ units per ml) coding for hChR2(H134R)- mCherry under the control of the promoter for hsyn was injected for 4 min and left the syringe in place for another 3 min, optic cannula was implanted above the LHb. Mice were set aside for at least two weeks of recovery and virus expression before performing CPP experiments. For retrobeads labelling (LumaFluor Inc.), mice were injected with fluorescent retrobeads (0.2 µl) in the LHb (AP: 1.7, ML: 0.45, DV: −2.8).

### Conditioned place preference (CPP)

The study utilized biased CPP training using the Med Associates CPP System and was conducted two weeks after virus injection to allow for channel rhodopsin expression.

On day 1, all mice underwent habituation in the CPP apparatus to determine their initial preference for compartments. The least preferred compartment was designated as the drug-paired chamber. Mice were then randomly assigned to the following four groups (1) Cocaine injection + optical stimulation, (2) Cocaine injection only, (3) Saline injection + optical stimulation, and (4) Saline injection only. Cocaine (15 mg/kg, i.p.) or saline (1 ml/kg, i.p.) injections took place in the appropriate chamber from day 2 to day 9, accompanied by optogenetic stimulation (20-Hz pulses, 15-ms pulse duration, 10 s/min) for mice in groups 1 and 3. The light stimulus intensity from the fiber tip (0.3 mW) was selected to reduce the probability of off-target activation of nearby thalamic fibers. On day 10, place preference tests were performed. Mice were placed in a neutral center chamber and allowed to move freely between the drug-paired and saline-paired chambers for 15 min. The time spent in each chamber was recorded using photobeam crossing and a CPP score was determined as follows:

$$CPP=\frac{Time\,in\,Chamber\,(group\,1,2\,or\,3)-Time\,in\,Chamber\,(group\,4)}{Time\,in\,Chamber\,(group\,1,2\,or\,3)+Time\,in\,Chamber\,(group\,4)}\times 100$$

### Immunohistochemistry

60 min following CPP test, mice were deeply anesthetized with isoflurane and trans-cardially perfused with phosphate-buffered saline (PBS) followed by 4% paraformaldehyde (Sigma, Germany) in PBS. Brains were then harvested and submerged in 4% paraformaldehyde overnight and transferred to 30% sucrose in ddH20. Thirty-micrometer Horizontal LHb-containing slices were obtained using the cryostat (Leica). Sections were immediately permeabilized and blocked in 0.3% Triton and 5% Normal Donkey Serum (NDS) in PBS for 1 h. For c-fos activity analysis of LHb cells, primary antibodies were added (NeuN 1:500, ab.104224, Abcam, and c-fos 1:2000, ab.226 308, Synaptic Systems) directly to the blocking solution and incubated at 4° overnight. The next day, sections were washed three times in PBS containing 0.3% Triton and 1% NDS. Sections were then transferred to washing solution containing secondary antibodies (1:500, Alexa flour 488; ab.150117, and cy5 1:500, ab.6564, Abcam) and incubated at room temperature for 2 h before washing three times in washing solution. Sections were mounted on Superfrost slides using IMMU MOUNT (Thermo scientific, USA). Images were acquired on a Fluoview FV10i confocal microscope using a 10× or 60× objective and analyzed using Olympus fluoviewer software and Nikon confocal microscope using NIS-Element’s viewer.

### Western blot analysis

Two hours following CPP test, mice were first anesthetized using isoflurane. Following anesthesia, they were decapitated, and their brains were immediately removed. Coronal sections containing the PFC, LHb, and VTA were obtained. These sections were then microdissected bilaterally on an ice-cold platform and promptly transferred to liquid nitrogen to preserve the tissue. The microdissected tissue samples were homogenized using a homogenization buffer consisting of 320 mM sucrose, 10 mM Tris-HCl (pH 7.4), 1 mM EDTA, 1 mM EGTA, a protease inhibitor cocktail (Sigma, P8340), and phosphatase inhibitors (1 mM Na_3_VO_4_ and 5 mM NaF by Sigma-Merck, Darmstadt, Germany). To determine the total protein concentration in the brain homogenates, the Pierce™ BCA Protein Assay Kit (Pierce, IL, USA) was utilized, with bovine serum albumin (BSA) as a standard. The protein samples were then boiled for 5 min at 95 °C to denature the proteins and loaded (20 µg protein /lane) onto 8–12.5% SDS-PAGE gels. Electrophoresis was performed to separate the proteins based on their molecular weight, and the separated proteins were transferred onto a nitrocellulose blotting membrane (TAMAR, Germany). Following the transfer, the membranes were incubated at room temperature for 1 h in a blocking buffer containing 5% non-fat dry milk. This step helps prevent nonspecific binding of antibodies. The membranes were then incubated overnight at 4 °C with the desired primary antibodies. The primary antibodies used in this study were Phospho ERK (Thr202/Tyr204) (cat. no. 91015; 1:1,500; Cell Signaling), ERK (cat. no. 9102s; 1:1000; Cell Signaling), GluR1 (cat. no. AB1504; 1:2,000; Merck), Anti-phospho-GluR1 (Ser845) (cat. no. AB 5849; 1:500; Merck), Anti-phospho-GluR1 (Ser831) (cat. no. 04-823; 1:500; Merck), and Beta Actin (cat. no. ab8227; 1:7,000; Abcam). After primary antibody incubation, the membranes were washed to remove unbound antibodies and then incubated with the appropriate HRP-conjugated secondary antibody for 1.5 h at room temperature. The secondary antibody recognizes and binds to the primary antibody, allowing for the detection of the target proteins. The blots on the membrane were detected and quantified using the Clarity™ western ECL system and the BioRad ChemiDoc™ XRS+ Imaging system (BioRad, CA, USA). This detection method utilizes chemiluminescence to visualize the bound antibodies on the membrane and quantify the protein bands.

### Statistical analysis

For behavioral analysis, IHC analysis two-way ANOVA was assessed to determine significant differences followed by post hoc Tukey honestly significant (HSD) tests when significant changes were found. For WB, analysis one-way analysis of variance (ANOVA) was assessed to determine significant differences, followed by post hoc Tukey honestly significant difference (HSD) tests when significant. Data are presented as mean ± SEM. All group sizes and significant differences are reported in the figure legends. The statistical analysis was performed using Prism 9.

## Results

### Optic stimulation of PFC-LHb neurons during conditioning abolished the expression of cocaine CPP

**Fig. 1:**
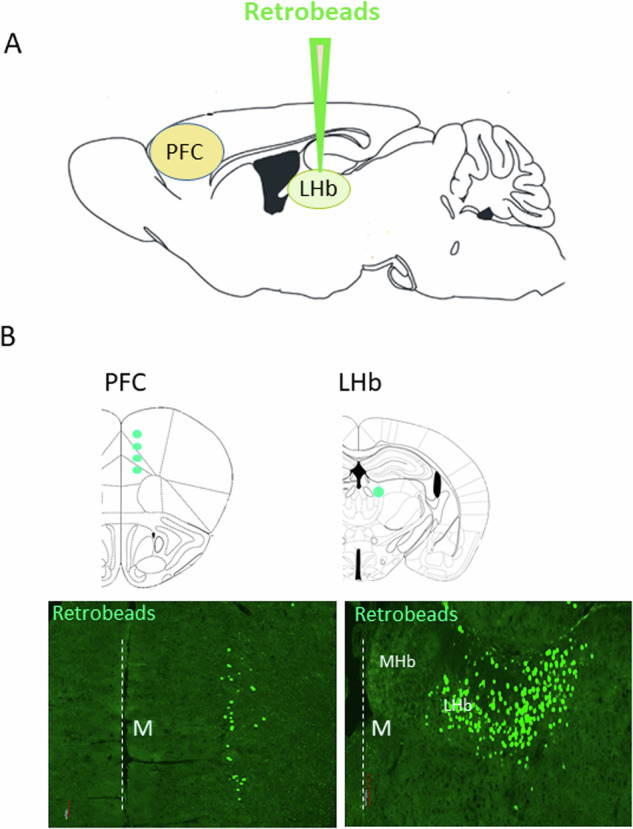
PFC projects to the LHb. **A** Retrobeads (green) were injected into the LHb. **B** Two weeks following injection, brains were analyzed for retrobeads signals. Fluorescent signals were observed in the PFC (left) and in the LHb (right), (M) midline is assigned with dashed line.

Previous studies have demonstrated that the LHb receives significant input from the PFC [9, 24]. Activation of these specific projections has been shown to impact social behaviors [6] and behavioral responses to challenging situations [5]. Given the pivotal role of the LHb in reward processing [7], and the substantial role of the PFC in the reward system, we first verified the presence of these projections. Therefore, we injected mice with green fluorescent retrobeads into the LHb (Fig. 1A) and assessed their distribution after a 2-week period. Retrograde labeling was observed in the LHb, as well as in specific PFC neurons (Fig. 1B), consistent with previous research findings [25].

**Fig. 2:**
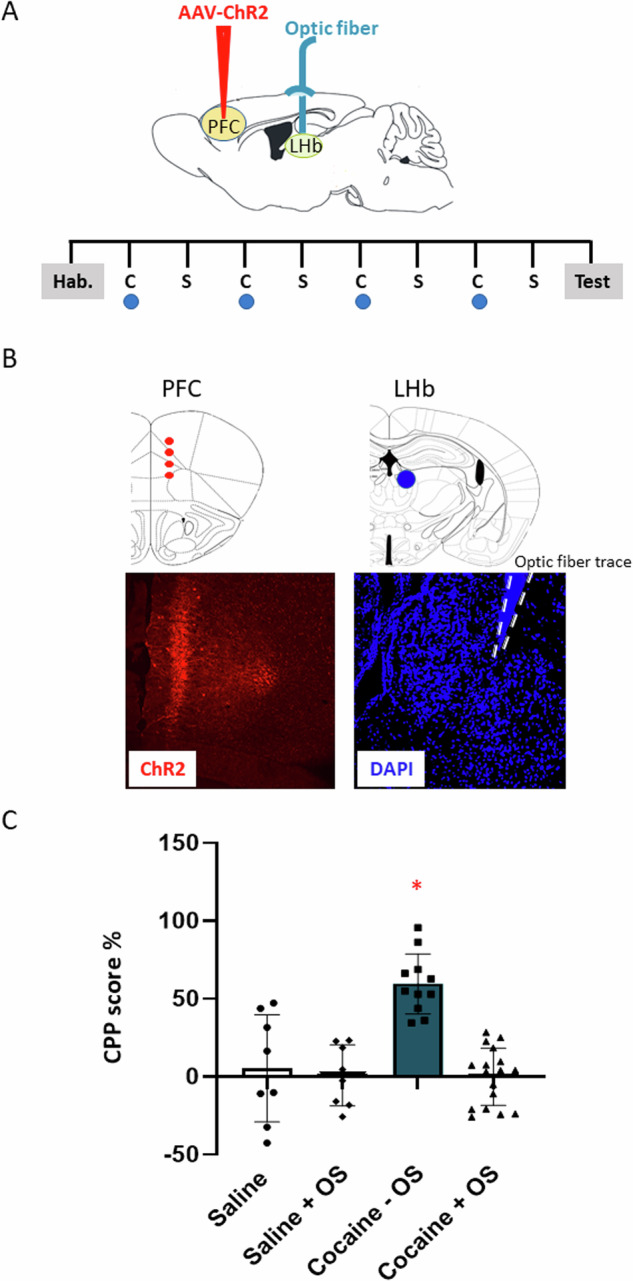
Optic stimulation of PFC-LHb neurons during conditioning abolished the expression of cocaine CPP. **A** AAV-ChR2 was injected in the PFC and optic fiber was implanted above the LHb. Two weeks post-injection of the AAV-ChR2 to the PFC, animals were introduced into the CPP apparatus for habituation session. The preference of each individual subject for a particular environment prior to conditioning was assessed. The least preferred compartment for each subject was then assigned to be the cocaine-paired compartment (biased) during conditioning. Following 8 days of conditioning (day 10), mice were tested for their preference to the cocaine-associated chamber. **B** AAV-ChR2 expression in the PFC (left) and optic fiber trace above the LHb (right, trace surrounded by dashed line). **C** After habituation, mice were divided into four groups, saline group without optic stimulation (saline, n = 8), saline group with optic stimulation in one chamber during conditioning (saline + OS, n = 8), cocaine group without optic stimulation during cocaine conditioning (cocaine –OS, n = 11) and test group, cocaine group that received optic stimulation during cocaine conditioning (cocaine + OS, n = 17). Graph depicting preference values expressed as mean CPP score ±SEM at test day. Cocaine – OS group, showed significant preference to cocaine-associated chamber and significantly differs from the three other groups (interaction factor, *p* = 0.0004, optic stimulation factor, *p* < 0.0001, treatment) (saline/ cocaine factor, *p* = 0.0005, two-way ANOVA, followed by post hoc Tukey HSD tests *p < 0.0001).

Recent research has demonstrated that the activation of distinct neurons in the PFC can lead to changes in specific behaviors. By utilizing optogenetic projection-targeting to control cells with precise efferent wiring patterns, it has been shown that selectively activating PFC neurons projecting to the LHb can have a profound, rapid, and reversible impact on the selection of the active behavioral state [5]. Therefore, we hypothesized that the exogenous activation of PFC-LHb projections during cocaine conditioning would influence cocaine preference in the treated animals. To test this hypothesis, we employed exogenous light activation of PFC-LHb projections (Fig. 2A, B) during cocaine exposure using the CPP protocol. Mice that received cocaine without optic stimulation exhibited a significant preference for the cocaine-associated chamber. However, when we activated PFC-LHb projections during cocaine conditioning, it abolished the preference for cocaine (Fig. 2C; interaction factor, (1, 40) = 15.19, *p* = 0.0004, optic stimulation factor, F(1,40) = 20.58, *P* < 0.0001, treatment (saline/ cocaine) factor, F(1,40) = 14.13, *p* = 0.0005, two-way ANOVA followed by post hoc Tukey HSD tests *p < 0.0001). Optic stimulation of PFC-LHb projections in saline-injected animals did not affect the animals’ preference, and no significant difference was observed between the saline group with optic stimulation and the saline group without optic stimulation (Fig. 2C, *p* = 0.9774, post hoc Tukey HSD tests).

### Optic stimulation of PFC-LHb projecting neurons during cocaine conditioning increased neuronal activity in the LHb

**Fig. 3:**
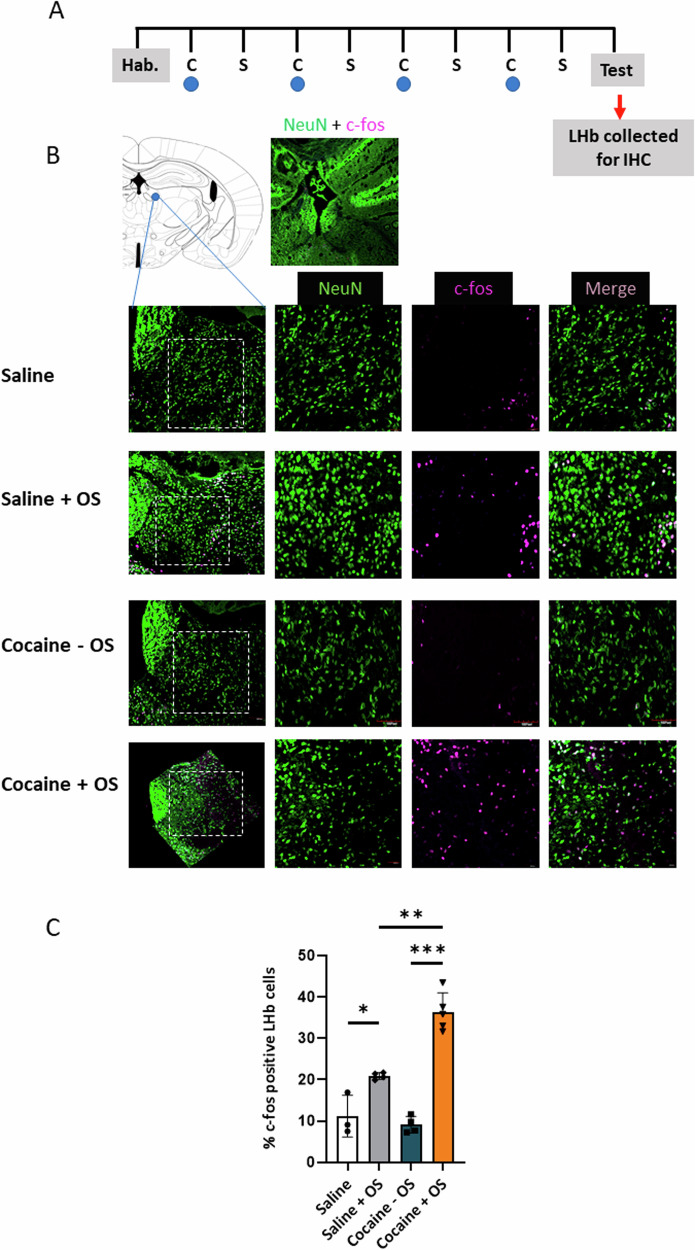
Optic stimulation of PFC-LHb projecting neurons during cocaine conditioning increased c-fos expression in the LHb following expression of CPP. **A** 24 h following cocaine/saline conditioning in the four mice groups (saline groups, n = 3, saline + OS group, n = 4, cocaine – OS group, n = 4, and cocaine +OS group, n = 5), cocaine preference was tested in the CPP apparatus, and 60 min after test mice brains were collected for IHC. **B** LHb-containing coronal slices were stained with NeuN (neuronal marker, green) and c-fos (neuronal activity marker, purple) antibodies. LHb (dashed square) displayed moderate c-fos expression in mice subjected to optic stimulation during saline conditioning (saline +OS group) while showing weaker expression in both the saline and cocaine - OS groups. Notably, the most robust c-fos expression was observed in the cocaine + OS group. **C** Graph depicting the percent of c-fos positive cells from the total LHb neurons expressed as mean ± SEM. The percentage of c-fos positive LHb cells was significantly higher in cocaine + OS group (interaction factor, *p* = 0.0004, two-way ANOVA). The percentage of c-fos positive LHb cells in saline + OS group is significantly higher from groups without optic stimulation (optic stimulation factor, *p* < 0.0001, two-way ANOVA, treatment factor (cocaine/ saline), *P* = 0.0029, two-way ANOVA). (*p = O.O164, **p = 0.0001, ***p < 0.0001, Post hoc Tukey HSD tests).

The LHb is a pivotal component in the brain’s response to reward [7]. Its activity is thought to encode the organism’s reward state, with diminished activity typically associated with a positive reward state and enhanced activity in response to a negative reward state [26]. Hence, we sought to investigate the changes in LHb neuronal activity following the activation of the PFC-LHb circuit during cocaine conditioning. Since the activation of PFC-LHb neurons during conditioning effectively abolished the expression of CPP, we postulated that optically stimulating PFC-LHb projecting neurons during conditioning would enhance LHb neuronal activity. To test this hypothesis, we employed the assessment of c-fos expression, a recognized marker for neuronal activity [27], in the LHb after the CPP test (Fig. 3A). During this test, which was conducted 24 h following the final conditioning session, the environmental cues have the capacity to elicit neuronal activity in various reward-related brain regions, even in the absence of cocaine. Mice that underwent optic stimulation of the PFC-LHb circuit during cocaine conditioning exhibited a significant increase in c-fos-positive neurons within the LHb (Fig. 3B, C), compared to animals that received cocaine or saline without optic stimulation. A two-way ANOVA analysis demonstrated a significant impact of optic stimulation during cocaine conditioning on cocaine preference (interaction, F (1, 12) = 23.72, *p* = 0.0004, optic stimulation factor, F (1, 12) = 105.9, *p* < 0.0001, treatment (cocaine/ saline) factor, F (1, 12) = 13.92, *P* = 0.029). To investigate whether the observed elevation in c-fos expression is linked to the elimination of cocaine preference or is solely a consequence of optic stimulation unrelated to cocaine treatment, we examined c-fos expression in mice subjected to optic stimulation during conditioning after receiving saline injections. It was evident that optic stimulation during saline conditioning induced a notable increase in c-fos expression compared to the saline group without optic stimulation (*p = 0.0164, Post hoc Tukey HSD tests). However, c-fos expression in the cocaine group with optic stimulation was significantly higher than that in the saline group with optic stimulation (**p = 0.0001, Post hoc Tukey HSD tests). These results strongly suggest that optic stimulation during conditioning leads to heightened neuronal activity in the LHb during cue exposure on the test day. Importantly, this effect is further amplified when optic stimulation is combined with cocaine exposure. This finding offers a plausible explanation for the prevention of cocaine preference observed in the cocaine + optic stimulation (OS) group.

### Optic stimulation of PFC-LHb projections during cocaine conditioning induces molecular changes in the PFC, LHb and VTA following test

**Fig. 4:**
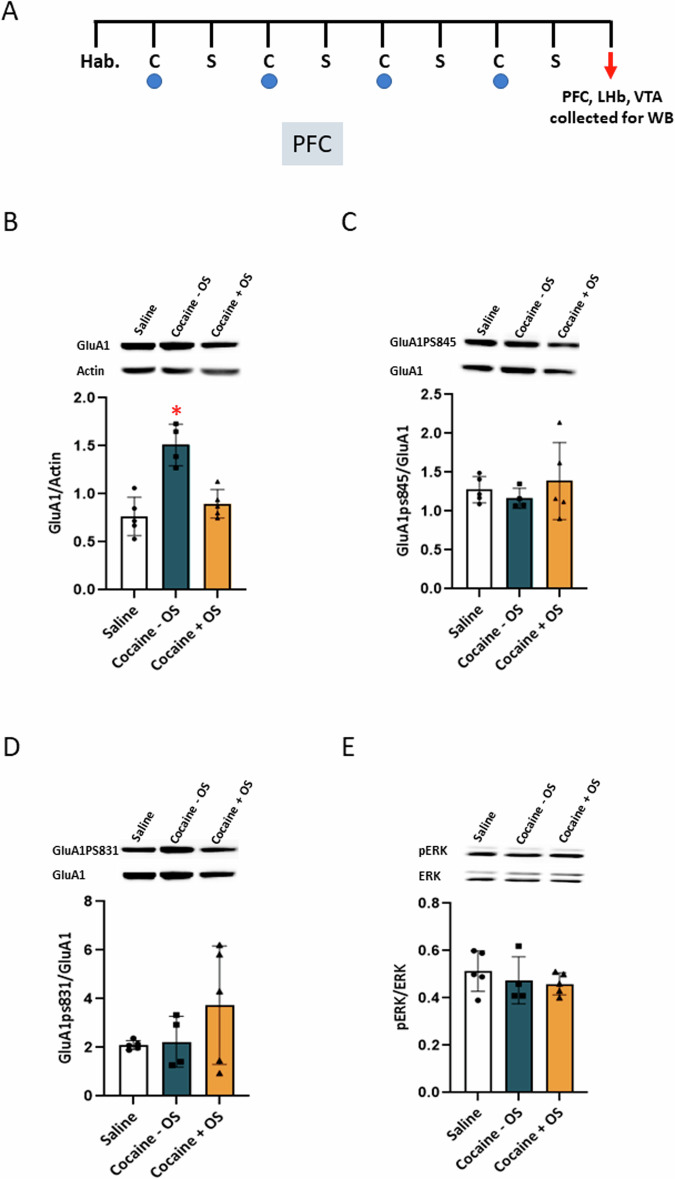
Optic stimulation of PFC-LHb projections during cocaine conditioning prevents the changes in GluA1 levels in the PFC. **A** 24 h following cocaine/saline conditioning for the three mice groups as in Fig. 4, cocaine preference was tested in the CPP apparatus, two hours after test, mice brains were collected for western blot analysis. **B** Optic stimulation of PFC-LHb projections during cocaine conditioning attenuated increased GluA1 expression in the PFC (saline group, n = 5, cocaine – OS group, n = 4, and cocaine + OS group, n = 5). The bar histograms depict the level of GluA1 divided by Actin, cocaine – OS showed a significant increased expression (p = 0.0003, one-way ANOVA). **C**, **D**, **E** No change was detected in GluA1ps845, GluA1ps831, and pERK (*p* > 0.05, one-way ANOVA).

We hypothesize that the activation of the PFC-LHb circuit, resulting in reduced preference for cocaine, would manifest as alterations in key synaptic proteins within the PFC, LHb, and VTA regions. This, in turn, could shed light on the underlying molecular mechanisms and signaling pathways involved. To explore this hypothesis, we collected samples from these brain regions two hours after the CPP test and conducted a western blot analysis (Fig. 4A). In our analysis, we focused on evaluating changes in the expression and phosphorylation state of two crucial proteins: The GluA1 subunit of the AMPA receptor and ERK (Extracellular Signal-Regulated Kinase). The GluA1 subunit of the AMPA receptor plays a pivotal role in mediating excitatory neurotransmission in the brain [13]. Phosphorylation of specific serine residues on the intracellular tail of GluA1, particularly S831 and S845, is known to be of critical importance in regulating the trafficking and function of GluA1. These phosphorylation events can influence the receptor’s localization to synapses and its overall synaptic efficacy [13, 28, 29]. ERK, a member of the MAPK signaling pathway, plays a vital role in various cellular processes, including neuronal plasticity. The phosphorylation state of ERK is often assessed to determine its activation state and its involvement in signaling pathways, which provides insights into its role in subsequent cellular events [30]. Two hours following the CPP test (Fig. 4A), we collected brain regions for analysis and conducted western blot assays to investigate these synaptic protein changes. In the cocaine-exposed group without optic stimulation, a noteworthy increase in GluA1 expression in the PFC was observed after the test (Fig. 4B), as confirmed by a one-way ANOVA (F (2, 11) = 19.22, *p* = 0.0003), and subsequently validated through Tukey’s multiple comparison test. Significantly, when PFC-LHb projections were optogenetically stimulated during cocaine conditioning, this context-induced elevation in GluA1 expression was substantially attenuated. No discernible alterations were observed in the levels of S831, S845, or pERK (Fig. 4C, D, F). It is worth mentioning that the time course for ERK phosphorylation is usually an increase within few minutes and falling to baseline within 20–45 min [31]. However, we have previously shown that after repeated cocaine exposure remained elevated one or even three weeks following withdrawal [28], although long-lasting changes in ERK phosphorylation can be a distinct process from acute ERK phosphorylation. Together, our results suggests that these events are highly depends on the mode of activation.

**Fig. 5:**
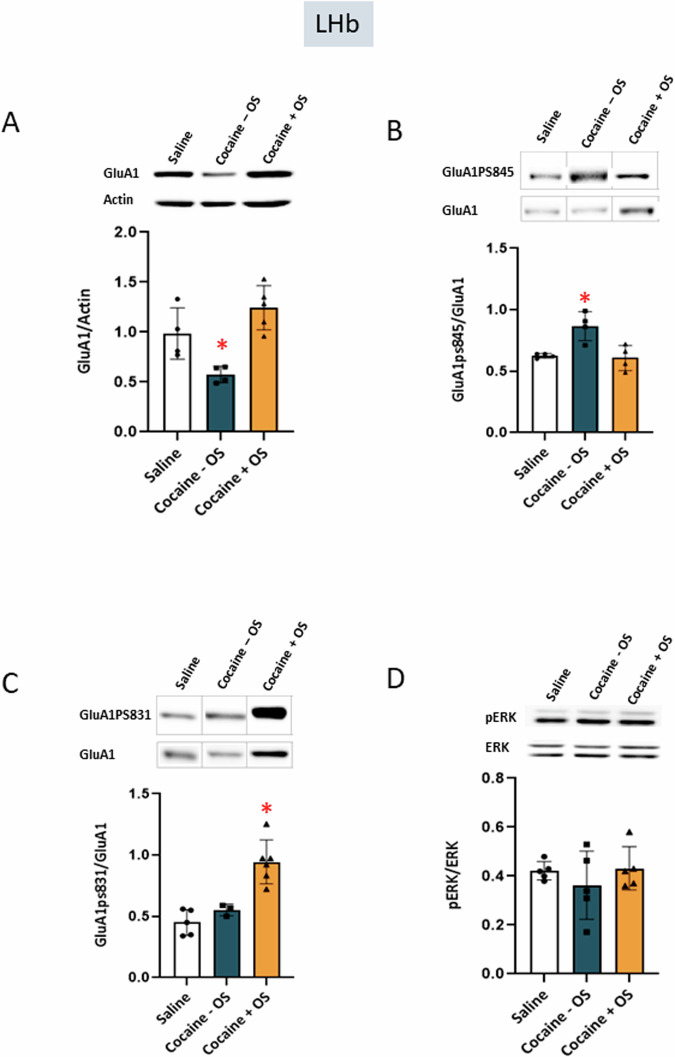
Optic stimulation of PFC-LHb projections attenuated changes in GluA1 and GluA1ps845 and caused increased GluA1ps831 levels in the LHb. **A** The bar histograms depict the level of GluA1 divided by Actin, cocaine – OS group showed significant decrease in expression levels (*p* = 0.0021, one-way ANOVA; saline group n = 4, cocaine – OS group n = 4, cocaine + OS group n = 5). **B** Optic stimulation prevents cocaine-induced increase in ps845 (Cocaine – OS; *p* = 0.0048, one-way ANOVA; saline group, n = 4, cocaine – OS group, n = 4, cocaine + OS group, n = 4). **C** Optic stimulation caused a significant increase in expression levels of ps831 (Cocaine + OS; *p* = 0.0003, one-way ANOVA; saline group n = 5, cocaine – OS group n = 3, cocaine + OS group n = 6). **D** No significant difference was found in pERK (*p* > 0.05, one-way ANOVA; saline group n = 5, cocaine – OS group n = 5, cocaine + OS group n = 5).

The LHb is a key player in regulating responses to cocaine, and exposure to this drug can diminish its regulatory function [32]. In our study, we selectively activated LHb neurons, resulting in heightened neuronal activity in the LHb following the CPP test. Consequently, we investigated the molecular changes in the LHb following this test. Our findings revealed that cocaine conditioning led to a significant decrease in GluA1 expression in the LHb after the test (Fig. 5A), as determined by a one-way ANOVA (F (2, 10) = 12.08, *p* = 0.0021), followed by Tukey’s multiple comparison test. Furthermore, cocaine conditioning induced a notable increase in GluA1pS845 expression in the LHb (Fig. 5B), one-way ANOVA (F (2, 9) = 10.21, *p* = 0.0048), as validated by Tukey’s multiple comparison test. Importantly, when we applied optogenetic stimulation of the PFC-LHb projections during cocaine conditioning, this significantly mitigated cocaine-induced alterations following test (Fig. 5A, B). Moreover, when optic stimulation was employed during cocaine conditioning, it led to a significant increase in GluA1pS831 expression in the LHb (Fig. 5C), as determined by a one-way ANOVA (F (2, 11) = 19.29, *p* = 0.0003), which was subsequently verified through Tukey’s multiple comparison test. Collectively, our results indicate that the activation of the PFC-LHb circuit during cocaine conditioning effectively mitigates the molecular alterations in GluA1 expression and GluA1pS845 induced by cocaine. Furthermore, the application of optical stimulation enhances the expression of GluA1pS831 in the LHb.

**Fig. 6:**
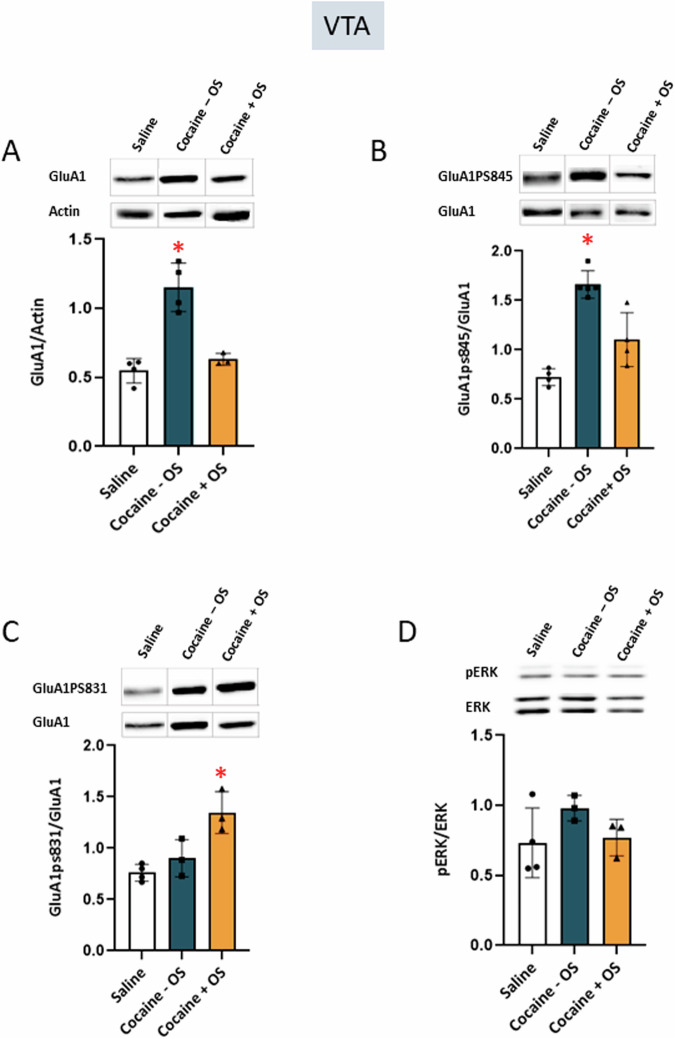
Optic stimulation of PFC-LHb projections during cocaine conditioning attenuated changes in GluA1 and GluA1ps845 expression and caused increased GluA1ps831 levels in the VTA after test. **A** Optic stimulation during cocaine conditioning attenuated the changes in GluA1 expression (Cocaine –OS; *p* = 0.0002, one-way ANOVA; saline group n = 4, cocaine – OS group n = 4, cocaine + OS group n = 3). **B** Optic stimulation during cocaine conditioning attenuated the changes in expression of GluA1ps845 (Cocaine – OS; *p* < 0.0001, one-way ANOVA; saline group n = 4, cocaine – OS group n = 5, cocaine + OS group n = 4). **C** Optic stimulation caused a significant increase in GluA1ps831 (Cocaine + OS; *p* = 0.0046, one-way ANOVA; saline group n = 4, cocaine – OS, n = 3, cocaine + OS n = 3). **D** No significant change was found in pERK (*p* > 0.05, one-way ANOVA; saline n = 4, cocaine – OS, n = 3, cocaine + OS n = 3).

Activation of the LHb robustly suppresses DA neurons in the VTA [7, 33]. It is suggested that the LHb regulation of VTA DA activity might underlie the aversive effects associated with the use of addictive substances [26, 34]. Therefore, we examine molecular changes in VTA following cocaine conditioning and PFC-LHb circuit activation. Cocaine conditioning induced a significant increase in GluA1 expression in the VTA after the test (Fig. 6A), as determined by a one-way ANOVA (F (2, 8) = 28.68, *p* = 0.0002), and subsequently verified through Tukey’s multiple comparison test. Additionally, we examined changes in GluA1 phosphorylation and found that conditioning led to a significant elevation in GluA1pS845 in the VTA (Fig. 6B), as determined by a one-way ANOVA (F (2, 10) = 31.14, *p* < 0.0001), and this was confirmed by Tukey’s multiple comparison test. The optogenetic stimulation of PFC-LHb projections effectively mitigated these changes in both GluA1 expression and GluA1pS845 following conditioning (Fig. 6A, B). Notably, optic stimulation during cocaine conditioning resulted in a substantial increase in GluA1pS831 expression in the VTA (Fig. 6C), as determined by a one-way ANOVA (F (2, 7) = 12.76, *p* = 0.0046), and this was confirmed by Tukey’s multiple comparison test. Taken together these findings highlight the significant role of the PFC-LHb pathway in modulating reward-related behavioral responses and in preventing the molecular changes in excitability and signaling induced by cocaine exposure.

## Discussion

This study investigates the role of PFC-LHb projections in the regulation of cocaine-conditioned reward, using cocaine place preference. Optogenetic stimulation of the PFC-LHb circuit during cocaine conditioning abolished the expression of cocaine preference. These observations are further supported by immunohistochemical analysis of neuronal c-fos expression in LHb neurons, where optic stimulation of the PFC-LHb circuit during conditioning led to increased neuronal activity in the LHb following the expression of CPP. Furthermore, western blot analysis in the PFC, LHb, VTA revealed that the stimulation of the PFC-LHb circuit during conditioning prevented or reversed the alterations in cocaine-induced expression of GluA1 and GluA1pS845. Additionally, it caused an increase in GluA1pS831 following the expression of CPP. These results suggest that activation of the PFC-LHb circuit during conditioning not only alters cocaine preference but also manifests by reversing the molecular changes in the expression and phosphorylation state of GluA1.

Human studies have suggested that deficits in prefrontal cortical function and the subsequent loss of inhibitory control could play a crucial role in promoting compulsive drug use [35, 36]. The LHb encodes negative reward signals that influence decision-making processes [7, 37]. While the LHb receives direct input from the PFC [38], little is known about the function of the PFC-LHb circuit in controlling drug reward and addiction. In our study, we hypothesized that the activation of PFC-LHb projections would negatively impact the behavioral-related rewarding properties of cocaine. We achieved this by utilizing optic stimulation during cocaine conditioning, with a deliberate choice of low LED intensity (0.3 mW) to minimize the likelihood of unintended activation of nearby thalamic fibers [6]. Our findings indicate that optogenetic stimulation of PFC-LHb projections during cocaine conditioning effectively prevented cocaine preference. Despite the LHb’s established role in processing aversive stimuli, our study demonstrates that the specific optic stimulation of the PFC-LHb circuit abolished cocaine-conditioned reward without inducing aversion [6], underscoring its precise and targeted action. Moreover, our results suggest that optical stimulation during the conditioning phase induces elevated neuronal activity in the LHb when subjects are subsequently exposed to the conditioning environment during CPP test. Notably, this increase in neuronal activity is further augmented when optical stimulation coincides with cocaine conditioning. This may elucidate the mechanism underlying the prevention of a cocaine preference in the group that received optical stimulation during cocaine conditioning.

Adaptations leading to addiction involve glutamate-dependent cellular mechanisms that facilitate learning and memory processes [39–41]. Addiction can be seen as a form of impaired neuronal plasticity [13]. Various brain regions exhibit differences in their responses to drugs of abuse and the underlying neuroplasticity mechanisms [42]. The AMPA receptor, an ionotropic glutamate receptor crucial for rapid excitatory synaptic transmission in the central nervous system, can be modulated during fundamental processes such as long-term potentiation (LTP) and long-term depression (LTD) [13]. Protein phosphorylation plays a pivotal role in regulating both ion channel properties and the synaptic targeting of GluA1-containing AMPA receptors during LTP and LTD [16]. In our study, we investigate changes in the phosphorylation of serine 845 (S845) and serine 831 (S831) on the GluA1 C-terminus. This phosphorylation critically controls the membrane delivery of AMPA receptors in various brain structures [13, 16, 29, 43]. Consistent with these prior studies, our findings indicate reduction in GluA1 levels and an increase in GluA1pS845 in the LHb following cocaine preference expression (at test day) in cocaine group. However, these alterations were mitigated by the activation of PFC-LHb circuit during the conditioning phase. A previous investigation explored the impact of cocaine on glutamatergic synaptic transmission onto LHb neurons projecting to the rostromedial tegmental nucleus (RMTg) in mice. This study revealed a significant elevation in S845 phosphorylation in LHb neurons projecting to the RMTg after cocaine treatment, with no observed changes in pS831 or total GluA1 levels [44]. These findings indicate that cocaine promotes the S845 phosphorylation-dependent delivery of GluA1 to LHb neurons projecting to the rostromedial tegmental nucleus (RMTg), which aligns with our observation of elevated GluA1pS845 levels resulting from cocaine conditioning. In our study, we assessed GluA1 levels across the entire LHb region and discovered a reduction in GluA1 expression following cocaine conditioning. This decline in GluA1 expression may imply a weakening of the synaptic strength of LHb neurons after exposure to cocaine conditioning. Furthermore, our findings indicate that optical stimulation during cocaine conditioning led to elevated levels of GluA1pS831. This increase in GluA1pS831, governed by the CaMKII (Calcium/Calmodulin-Dependent Protein Kinase II) pathway, suggests that AMPA receptors containing these phosphorylated GluA1 subunits exhibit heightened channel conductance [23]. This augmentation in channel conductance may contribute to the reinforcement of excitatory synaptic transmission in the LHb. Furthermore, the phosphorylation of GluA1 at serine 831 has been linked to the synaptic incorporation of AMPA receptors [13, 16]. Therefore, the elevated GluA1pS831 levels are likely to contribute to the insertion of GluA1-containing AMPA receptors into the synapses of LHb neurons. This could potentially account for the increased levels of GluA1 observed following cocaine conditioning when combined with optical stimulation of the PFC-LHb circuit. Based on our findings, we hypothesize that the heightened GluA1pS831 levels and subsequent insertion of GluA1-containing AMPA receptors result in the strengthening of synapses in the LHb. The increased neuronal activity, as indicated by heightened c-fos expression in the LHb following cocaine CPP, further reinforces our hypothesis.

In the VTA, cocaine induced an increase in GluA1 and GluA1pS845 levels, while the application of optical stimulation to the PFC-LHb circuit during conditioning mitigated this increase and resulted in elevated GluA1pS831 levels. Notably, the increase in GluA1pS831 levels in the cocaine + OS group was accompanied by a decrease in total GluA1 levels. It has been extensively documented that phosphorylation of AMPAR-GluA1 leads to its stabilization, increased channel conductance, and enhanced surface expression [45]. However, a recent discovery identifies S-nitrosylation of GluA1 at residue C875 as an additional pathway for modifying the AMPAR subunit, promoting phosphorylation at the same GluA1-S831 residue. This, in turn, leads to subsequent endocytosis of AMPA receptors in cortical neurons, thereby regulating synaptic plasticity [46]. We postulate that the increased levels of GluA1pS831 in the VTA, induced by optical stimulation of PFC-LHb projections during cocaine conditioning, downregulates GluA1 via this pathway [29]. However, since phosphorylation of GluA1-S831 was accompanied with a decrease in total GluA1 levels, it is possible to assume that the increased phosphorylation (calculated by GluA1-p831/GluA1) is not representing an increase but rather it’s a result from decrease in total GluA1. However, further research should be conducted in order to determine the precise molecular mechanism underlying intracellular trafficking of GluA1 during cocaine exposure or following stimulation.

In our experiments, optical stimulation of the PFC-LHb circuit activated terminals in the LHb. We have also found an increased expression of GluA1 in the PFC following the CPP test, and this increase was attenuated in the cocaine + OS group by the activation of PFC-LHb projections during cocaine conditioning. This observation can be explained by the fact that the PFC receives direct projections from dopaminergic neurons located in the VTA [47], in turn, heightened activity of VTA DA neurons enhances the excitability of PFC neurons [48]. Therefore, the increased activity in the VTA may provide an explanation for the elevated expression of GluA1 in the PFC at test day following cocaine conditioning. The heightened GluA1 levels in the PFC were reduced in the cocaine + OS groups when PFC-LHb projections were activated during conditioning. This activation appeared to have a suppressive effect on the VTA. Thus, in line with our initial explanation linking increased GluA1 levels in the PFC to heightened VTA activity, the decline in GluA1 levels in the PFC after optical stimulation during conditioning can be attributed to the reduced activity in the VTA.

It is worth mentioning that these experiments did not include saline + optic stimulation group and their biochemical analysis, since no behavioral change in CPP score was found in this group. However, we have recently shown that optic stimulation of RMTg-VTA did not cause any changes in the phosphorylation state of GluA1 in all regions that were tested [49], although a different population of neurons and different brain region was tested. In addition, recent neuroimaging studies have shed light on some of gender-specific differences. They have shown structural variances between male and female brains, both after acute and chronic exposure to substances like cocaine. Intriguingly, these studies have revealed opposite patterns of activity in key brain regions among males and females [50]. Therefore, it is important to conduct our future experiments comparing female to male mice.

By demonstrating that the activation of the PFC-LHb circuit during cocaine conditioning eliminates cocaine preference, our study underscores the potential of targeting this circuit for therapeutic interventions. Moreover, the insights related to GluA1 and its phosphorylation state in the LHb, VTA, and PFC offer vital information regarding the molecular mechanisms underline cocaine-induced neuroadaptations. Nonetheless, the precise manner in which optic stimulation-induced alterations in cocaine preference remains elusive. This phenomenon could conceivably be linked to a spectrum of factors, including potential modifications in the processing of reward signals, the induction of aversive learning responses, or even shifts in the intricate processes governing decision-making. Therefore, we cannot rule out the possibility that this manipulation has the potential to influence the rewarding properties of cocaine and not necessarily on learning and memory. A comprehensive understanding of these underlying mechanisms necessitates further exploration and investigation.

## Data Availability

The dataset generated and analysed during the current study are available from the corresponding author on reasonable request.
